# Draft genome of heterotrophic bacteria *Sphingomonas* sp. 2R-10 isolated from water treatment plant in South Africa

**DOI:** 10.1128/MRA.00437-23

**Published:** 2023-08-16

**Authors:** Rinaldo K. Kritzinger, Lesego G. Molale-Tom, Oluwaseyi Samuel Olanrewaju, Cornelius C. Bezuidenhout

**Affiliations:** 1 Unit for Environmental Sciences and Management, Potchefstroom Campus, North-West University, Private Bag, Potchefstroom, South Africa; Montana State University, Bozeman, Montana, USA

**Keywords:** *Sphingomonas*, genome analysis

## Abstract

The availability of clean drinking water is crucial for human well-being, necessitating the monitoring and characterization of microorganisms in water sources. In this study, we present the draft genome of a heterotrophic bacterium, *Sphingomonas* sp. 2R-10, isolated from the untreated raw water of a drinking water source in South Africa.

## ANNOUNCEMENT

*Sphingomonas*, a diverse and ubiquitous group of Gram-negative bacteria, holds immense ecological, environmental, and industrial relevance. They can be found in soil, water, and other natural environments (1, 2).

*Sphingomonas paucimobilis*, a species of *Sphingomonas*, has been discovered to cause infections in humans, particularly in immunocompromised persons (3). Milder skin infections to more dangerous bloodstream infections are all possible. Other rare and opportunistic *Sphingomonas* pathogens have been reported in various studies (4 - 6).

The *Sphingomonas* sp. 2R-10 was isolated from the influent in a water distribution source in North-West Province, South Africa, in August 2016. Strain isolation was performed on nutrient agar at 37°C for 24 h. A single colony was picked and streaked onto nutrient agar to obtain a pure isolate. The DNA was subsequently extracted using the chemagic DNA Bacteria Kit (PerkinElmer, Germany), following manufacturer’s instructions.

DNA concentration was quantified using a Nanodrop 1000 spectrophotometer (Thermo Fisher Scientific, USA). The DNA library was prepared using the Nextera XT Kit (Illumina Inc., San Diego, CA), according to the manufacturer’s instructions. The sequencing was performed on a MiSeq Illumina platform (Illumina Inc., CA, USA) at the North-West University sequencing facility. The generated raw paired-end fastq reads (2  ×  300 bp) were quality checked using FastQC v.0.11.7 (7) followed by trimming of low-quality bases using Trimmomatic v.0.39 (8), and the data quality was checked again using FastQC v.0.11.7 (7). The cleaned reads were assembled using SPAdes v.3.15.5 (9). To evaluate the quality of the genome assembly, Quast (v.5.0.2) (10) was used, and completeness and contamination were assessed using CheckM (v.1.1.6) (11) and the NCBI Prokaryotic Genome Annotation Pipeline v.6.5 (12). The annotated genomes were assessed against the Genome Taxonomy Database (GTDB) using GTDB-Tk software version 1.7.0 (13), and the result is shown in Table 1. Default parameters were used for all software except where otherwise noted.

**TABLE 1:** GTDB-TK classification report identifying the isolate as *Sphingomonas*

User genome	2R-10
Classification	d__Bacteria;p__Proteobacteria;c__Alphaproteobacteria;o__Sphingomonadales;f__Sphingomonadaceae;g__Sphingomonas;s__Sphingomonas sp003946805
FastANI reference	GCF_003946805.1
FastANI reference radius	95
FastANI taxonomy	d__Bacteria;p__Proteobacteria;c__Alphaproteobacteria;o__Sphingomonadales;f__Sphingomonadaceae;g__Sphingomonas;s__Sphingomonas sp003946805
FastANI ANI	100
FastANI alignment fraction	0.99
Closest placement reference	GCF_003946805.1
Closest placement taxonomy	d__Bacteria;p__Proteobacteria;c__Alphaproteobacteria;o__Sphingomonadales;f__Sphingomonadaceae;g__Sphingomonas;s__Sphingomonas sp003946805
Closest placement ANI	100
Closest placement alignment fraction	0.99
MSA AA percent	97.76

Based on CheckM, the genome completeness and contamination are 99.41 and 1.02, respectively. The genome revealed a total of 4,196,195 bp, 96 contigs, 67.5% GC content, N50 and L50 of 82,684 and 18, respectively, with a coverage of 76.0×. The prediction of gene functions revealed a total of 3,843 coding genes and 3,902 coding DNA sequences, 4 rRNAs, 50 tRNAs, and 2 CRISPR arrays.

In conclusion, this genome report will help in providing more insight into adaptation mechanisms, ecological roles, biotechnological applications, industrial implications, and functional annotations of *Sphingomonas* species. This report serves as a valuable resource for the field of microbial genomics.

## Data Availability

The whole-genome shotgun project for *Sphingomonas* sp. 2R-10 has been deposited at DDBJ/ENA/GenBank under the accession JASCXF000000000, and the version described in this paper is version JASCXF010000000. The raw reads are available under the BioProject accession number PRJNA968035, and the BioSample accession number is SAMN34894826. The sequence data obtained in this work have been deposited in the NCBI Sequence Read Archive under the accession number SRR24491037.
